# Controlling Light in Scattering Materials for Volumetric Additive Manufacturing

**DOI:** 10.1002/advs.202105144

**Published:** 2022-05-18

**Authors:** Jorge Madrid‐Wolff, Antoine Boniface, Damien Loterie, Paul Delrot, Christophe Moser

**Affiliations:** ^1^ Laboratory of Applied Photonics Devices School of Engineering Ecole Polytechnique Fédérale de Lausanne Lausanne Switzerland; ^2^ Readily3D SA EPFL Innovation Park, Building A Lausanne Switzerland

**Keywords:** bioprinting, complex materials, light‐based 3D printing, light scattering, volumetric additive manufacturing

## Abstract

3D printing has revolutionized the manufacturing of volumetric components and structures in many areas. Several fully volumetric light‐based techniques have been recently developed thanks to the advent of photocurable resins, promising to reach unprecedented short print time (down to a few tens of seconds) while keeping a good resolution (around 100 μm). However, these new approaches only work with homogeneous and relatively transparent resins so that the light patterns used for photo‐polymerization are not scrambled along their propagation. Herein, a method that takes into account light scattering in the resin prior to computing projection patterns is proposed. Using a tomographic volumetric printer, it is experimentally demonstrated that implementation of this correction is critical when printing objects whose size exceeds the scattering mean free path. To show the broad applicability of the technique, functional objects of high print fidelity are fabricated in hard organic scattering acrylates and soft cell‐laden hydrogels (at 4 million cells mL^−1^). This opens up promising perspectives in printing inside turbid materials with particular interesting applications for bioprinting cell‐laden constructs.

## Graphical abstract

Light‐based 3D printing allows for the fast and precise fabrication of structures with high geometrical complexity. However, these new approaches only work with transparent resins, so that the light patterns used for photo‐polymerization are not scrambled. Here, a method that boosts certain spatial frequencies is used to fabricate complex geometries in acrylates with TiO_2_ nanoparticles and in cell‐laden hydrogels.

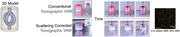

## 1 Introduction

Until only a few years ago, the conventional approach in light‐based additive manufacturing, or 3D printing, relied on constructing objects by piling 1D voxels or 2D layers on top of each other. Each layer being formed by the solidification of a photoresist under light irradiation, for example, by either scanning a laser beam point‐by‐point, namely stereolithography (SLA)^[^ ^1^ ^]^ or two‐photon fabrication,^[^ ^2^, ^3^ ^]^ or by projecting 2D light patterns, namely digital light processing (DLP) technology.^[^ ^4^, ^5^ ^]^ Recently, several new techniques for the additive manufacturing of photo‐sensitive materials have been proposed, including the holographic display of light patterns,^[^ ^6^ ^]^ tomography,^[^ ^7^, ^8^, ^9^ ^]^ and xolography.^[^ ^10^ ^]^ They derive from the layer‐by‐layer process by fabricating centimeter‐scale objects in a true 3D fashion. This is achieved by illuminating the entire resin's container with one or a set of light patterns used for photo‐polymerization. The cumulative light exposure results in a volumetric energy dose that is sufficient to solidify the material in the desired geometry. The main advantages of these so‐called volumetric additive manufacturing (VAM) approaches over existing sequential methods are their short building time (down to a few tens of seconds compared to tens of minutes for sequential approaches), and their ability to print complex hollow structures without the need for support struts. However, to achieve a correct 3D light dose deposition in the material, the light patterns used for photo‐polymerization must propagate inside the resin without being distorted or attenuated through the entire build volume. Although VAM has been used to fabricated pieces in acrylates,^[^ ^7^, ^9^ ^]^ polymer‐derived ceramics,^[^ ^11^ ^]^ thiol‐ene resins,^[^ ^12^, ^13^ ^]^ mixed acrylate and epoxy monomers,^[^ ^14^ ^]^ and cell‐laden hydrogels;^[^ ^8^ ^]^ its application has been confined to materials that exhibit high optical transparency and very little light scattering or absorbance. Tuning the optical properties of hydrogels with contrast agents to reduce the refractive index mismatch between cells and their medium can improve print fidelity, but it comes at the cost of changing the hydrogel composition.^[^ ^15^, ^16^ ^]^

We propose a method for a significant improvement of both printing fidelity and resolution of VAM, with a motivation to print in a scattering medium such as highly loaded cell‐laden hydrogels. In the present contribution, we apply it to tomographic volumetric printing in scattering acrylics and cell‐laden hydrogels, but our approach should also be applicable to other type of light‐based 3D printers and materials. The idea relies on first experimentally characterizing the propagation of light through the scattering resin and then iteratively correcting the projected patterns displayed onto a digital micromirror device (DMD, 8‐bit depth amplitude modulation). We propose here to compensate for scattering by correcting not only the attenuation of ballistic light but also the increased blur of the pattern with depth. We demonstrate the technique's performances through a series of different centimeter‐scale printed objects in resin formulations whose scattering mean free path is around 5 mm.

Tomographic additive manufacturing consists of illuminating a volume of photo‐responsive material with a set of light patterns modulated in amplitude and projected from multiple angles. This technique enables printing centimeter‐scale objects with a typical resolution of up to 80 μm.^[^ ^7^, ^9^ ^]^ As any projection‐based printing system, the final resolution of the printed structure is theoretically determined by the effective pixel size of the DMD at the center of the build volume. Gray‐scale tomographic reconstruction algorithms have been used to improve print resolution^[^ ^12^, ^17^ ^]^ or print stiffness.^[^ ^14^ ^]^ In practice, the final resolution of the prints is also affected by the polymerization process of the resin and potential optical aberrations inherent to the experimental setup.^[^ ^9^, ^18^, ^19^ ^]^ Most problematically, light may also be scattered by the material itself. This is the case for all nontransparent materials: biological inks, hydrogels, or composite resins that are of utmost interest for many applications including bioprinting,^[^ ^20^ ^]^ medical devices,^[^ ^21^ ^]^ customized implants,^[^ ^22^ ^]^ and even jewelry that are all challenging to print with light‐based systems.^[^ ^23^, ^24^, ^25^ ^]^ When the light patterns propagate through such complex materials, they get optically distorted, and the spatial information they carry is scrambled. In addition, the intensity of ballistic light that is not deviated from its original path is attenuated exponentially with depth (Beer–Lambert law). Its characteristic distance is merely the scattering mean free path, denoted *l*_s_, and corresponds to the average distance between two successive scattering events. Both attenuation and distortion of the light patterns with depth corrupt the formation of the desired light dose in the material. This directly translates into a poor printing fidelity and a global loss of resolution that usually make the object nonfunctional. There is a need for taking into account the scattering properties of the resins when computing the projected light patterns.

## 2 Methods

**Figure 1:**
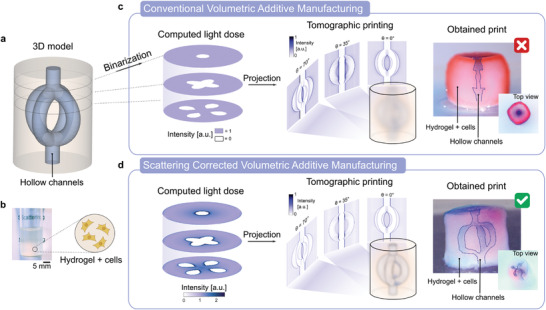
Scattering‐corrected tomographic volumetric additive manufacturing (VAM) allows to print complex geometries with hollow channels in scattering materials, such as cell‐laden hydrogels. a) A 3D model of an object with a core surrounded by four interconnected hollow channels. b) Example of a hydrogel containing 4 million cells mL^−1^. The text written behind a typical vial used for printing is not readable due to light scattering by the cells in suspension. c) In conventional tomographic VAM, the 3D model is binarized into a computed light dose that is used to calculate the set of patterns for printing. When these patterns are projected onto the scattering material, they are blurred and the resulting deposited light dose prevents from printing the target object. d) In scattering‐corrected VAM, the 3D model is transformed into a continuous light dose that accounts for light distortions by the gel. The projection of the corresponding light patterns produce a print that matches the geometry and the function of the target 3D model.

In tomographic VAM the light patterns are, in principle, only determined from the object's 3D shape. As described in ref. [7, 9] the conventional approach consists of, first, converting the target 3D model into a 3D binary matrix of voxels, where the entries “1” indicate the presence of matter and “0” its absence at each particular location in space. This matrix also represents the normalized target dose that one would need to deposit in a transparent resin to polymerize it in the desired geometry. A series of dose projections over multiple angles are calculated from the Radon transform as developed for computed tomography.^[^ ^26^ ^]^ More precisely, the patterns are obtained using a filtered back‐projection algorithm followed by an optimization subject to positivity constraint (see Section S1, Supporting Information). But, this forward model assumes that light is neither attenuated nor distorted along its propagation, which is no longer valid in turbid materials. For improving printability in nontransparent materials with the same apparatus, one has no other option than to optimize the set of projected patterns. Importantly, the patterns must not be modified individually, but rather as a whole set so that the results of their backprojections over one rotation fits the corrected dose that takes into account light scattering (**Figure** **1**d). To emphasize the importance of the scattering correction on the final print we report on the tomographic VAM of hydrogels containing 4 × 10^6^ human embryonic kidney 293 cells mL^−1^ (Figure 1). Here, the HEK 293 cells in suspension play the role of optical scatterers as their refractive index does not match exactly the one of the gel. The target object is a model of vasculatures which is, as any hollow structure, challenging to print. Optical scattering makes it difficult for light to reach the middle of the vial without over‐polymerizing the outer cylinder. As one can see on the Figure 1, without correction on the projected patterns (Figure 1c), it is impossible to print correctly the four channels: the functionality of the part is lost. By contrast, when the appropriate scattering correction is applied, the 3D dose deposited inside the vial is less affected by scattering and the four channels are thus well printed, as one can notice on Figure 1d.

**Figure 2:**
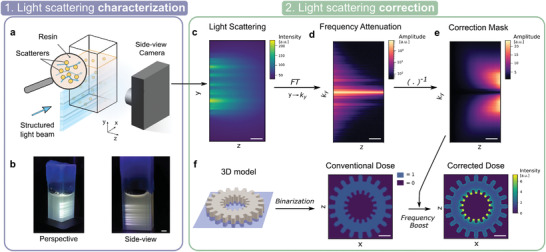
Light scattering is firstly characterized and secondly corrected. a) To characterize light scattering, a thin structured beam of light is projected across the resin while a side‐view camera records pictures. b) Photographs of a 10 mm‐thick cuvette used for the characterization. c) To correct for light scattering in the photocurable resins, images for different incoming light structures are captured by the side‐view camera. d) The Fourier transform of this dataset shows that high spatial frequencies are, in average, attenuated more rapidly with depth. e) By inverting this attenuation map in frequency space, we obtain a correction mask. This correction mask indicates how to boost spatial frequencies at different depths to counterbalance this effect of scattering. f) To print an object in a scattering resin, the 3D model is binarized and the correction mask is applied onto it. Scale bars = 2 mm.

The scattering correction is the key point for improving the printability of tomographic VAM in nontransparent resins. The level of correction depends on the amount of scattering, which is related to the concentration of scatterers but also their shape, size, and refractive index. Although these parameters can be theoretically estimated and then used to roughly approximate the amount of scattering in the sample, we propose to experimentally measure it in the printer. As depicted in **Figure** **2**a,b, this optical characterization consists of projecting one or a set of incoherent light patterns through the scattering resin using the DMD of the tomographic printer. The light patterns chosen for the scattering characterization are deliberately narrow along the *x*‐axis in order to improve the optical sectioning and increase the contrast of the camera image. The position of the cuvette is such that the projected patterns fall close to its edge. A camera, orthogonal to the optical axis, is used to capture side‐view images of the cuvette's lateral facet. The exemplary image recorded with the camera in Figure 2c shows that scattering results in (i) an exponential decrease of ballistic light with depth and (ii) an increased blur of the light pattern along its propagation. The fact that light patterns get increasingly blurred with depth is also noticeable in the frequency domain (namely *k*‐space, Figure 2d). The scattering material acts as a low‐pass filter. In other words, features of high spatial frequencies in the pattern get more rapidly attenuated than low‐spatial frequency features. In order to properly characterize the transmission of all the spatial frequencies, a sequence of different patterns is projected by the DMD onto the cuvette. Suitable sets of patterns include patterns from the Radon transform of the object to print, random patterns, or designed dictionaries of patterns with representative spatial frequencies, for example. In Figure 2d, the average transmission of the spatial frequencies over 100 different patterns highlights the strong attenuation of the high spatial frequencies as light penetrates in the material. To alleviate this unequal impact of scattering in *k*‐space, the amplitude of higher frequency components should be enhanced. According to these measurements, a correction mask is computed so that the amplitude of the frequency components is, in average, maintained at all depths *z* (Figure 2e). In practice, this correction mask is obtained by dividing the incident averaged spectrum at *z* = 0 mm by each spectrum taken at different depths. The correction mask is then applied onto the target binary map, conventionally used as 3D target dose to compute the patterns. In our tomographic system, the penetration depth increases radially and is maximal at the center of the vial due to its rotation (i.e., 8 mm in our case). This region is where the light scattering causes the highest distortion for printing. Therefore, it is also where the correction is the strongest, as one can see in Figure 2f. Compared to the binary map, this corrected dose computed from experimental measurements has a much higher contrast, especially when moving toward the center. Further information on how the correction is first measured and then applied are provided in Section S2, Supporting Information.

## 3 Results

We experimentally studied the performances of the method through the printing of different objects in two different scattering materials, an organic acrylate and a soft hydrogel. For both, we compare obtained 3D printed objects with and without the scattering correction. Importantly, except for the correction applied on the target dose, the procedure to compute the light patterns based on the Radon transform is the same (Section S1, Supporting Information).

**Figure 3:**
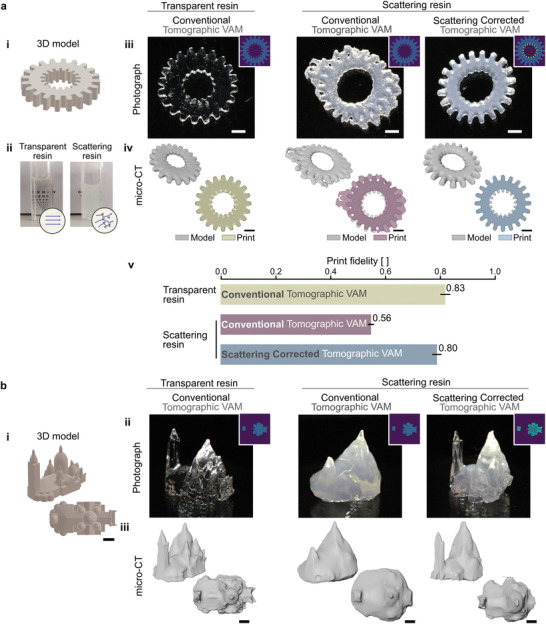
Scattering corrected tomographic VAM increases print fidelity. a) (i) The 3D model of a gear with cogs on its outer and inner circumferences is used as a target for assessing print fidelity. (ii) Photographs of the transparent and the scattering organic acrylate photocurable resin used for printing. Random mixing of light by TiO_2_ nanoparticles dispersed in the scattering resin, prevents from reading the text placed behind the 10 mm thick cuvette. Objects are printed in the transparent resin using conventional tomographic VAM and in the scattering resin using conventional and scattering‐corrected tomographic VAM. (iii) Photographs (insets show transversal light dose profiles) and (iv) micro‐CT scans of the resulting prints. (v) Print fidelity measured as the intersection over union (IoU) of the model and the print (IoU = 1 means perfect matching). Error bars indicate standard error. b) Scattering corrected tomographic VAM of complex 3D structures. (i) 3D model of the Sacré‐Cœur Basilica in Paris. (ii) Photographs (insets show transversal light dose profiles) and (iii) micro‐CT scans of the resulting prints. Scale bars = 2 mm

We use an organic resin with a pentaacrylate as a backbone in which a controlled amount of TiO_2_ nanoparticles is homogeneously dispersed as a scattering agent (**Figure** **3**a(ii) and Section S3, Supporting Information, see Experimental Section). This protocol allows us to increase the amount of scattering to ensure that it is deleterious for volumetric printing. First we use this resin to assess the gain in print fidelity. For this purpose we use the 3D model of a gear with inner and outer cogs as a target for print fidelity (Figure 3a(i)). These features are challenging to print because of their small size (inner cogs: width of 460 μm, outer cogs: width of 750 μm) and their position in the vial far close to the center, where light is mostly scattered (*l*_s_ = 6.1 mm, Section S3, Supporting Information). If no correction is applied, the only way to deposit more light in the vial's axis and thus print the inner teeth is to increase the dose. This can be done either by enhancing the laser power or by printing over a longer time. While such increase brings more light at depth, it also overexposes parts close to the wall of the vial. It results that when the inner cogs start to form, the outer ones are already over‐polymerized. It is in this precise situation that the correction intervenes to limit the damaging effects of scattering on the print. Instead of computing the light patterns from the binary dose, we use the target dose reconstructed from the experimental characterization of light scattering. Corresponding printed gears reported in Figure 3a(iii,iv), show the inner cogs are better defined and no over‐polymerization of the outer structure is observed. From this 2D+1 object, we can compute the intersection over union (IoU) of its *y*‐projection (Figure 3a(iv)),^[^ ^27^ ^]^ which is a quantitative metric of the print fidelity. ^[^ ^28^ ^]^ We report improvements in print fidelity from IoU =0.56 ± 0.02 to IoU =0.80 ± 0.03 by printing with a set of corrected tomographic patterns. The baseline print fidelity for this shape in a transparent resin was IoU=0.83 ± 0.03 (Figure 3a(v)). More importantly, applying corrections for scattering allows to fabricate a functional part with protrusions and indentations.

In Figure 3b, we report on the fabrication of a complex volumetric object: the Sacré Cœur Basilica in Paris. As in Figure 3a, we compare the prints obtained in a transparent acrylic with those obtained in its scattering counterpart (*l*_s_ = 4.8 mm) with and without scattering corrections. The correction applied onto the target 3D dose is done following the same protocol as in Figure 2. Cross‐sections along the *y*‐axis of the computed light dose show that more light is required on the edges of the object (regions of high spatial frequency) and deeper into the vial to counterbalance the effect of scattering (Figure 3b(ii)). This correction of the target dose translates into modifications of the set of projected patterns (see Section S4, Supporting Information). Figure 3b(ii,iii) are photographs and X‐ray scans of the obtained prints, respectively. As observed with the gear in Figure 3a, light scattering prevents from printing the full object without over‐polymerizing some parts of the structures, generally the outer ones. As one can see from Figure 3b(ii,iii) (middle panel), the main square tower cannot be printed correctly. This issue does not occur when the scattering is taken into account prior to computing the light patterns. Additionally, the general method can be applied to the particular case of highly absorptive resins, for instance when the concentration of photoinitiator is increased (Sections S5 and S6, Supporting Information).^[^ ^11^ ^]^

**Figure 4:**
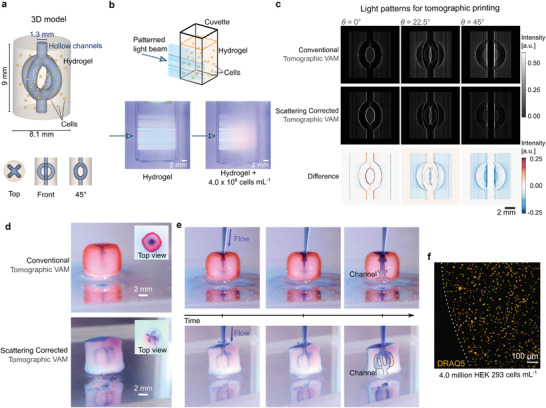
Bio‐fabrication of a functional vascular model in cell‐laden hydrogels. a) 3D model of a construct with a core surrounded by four channels, emulating vasculature. b) Side‐view of how light is blurred as it penetrates into the hydrogel with cells. c) Corresponding light patterns projected at different angles during tomographic VAM with and without correction. The difference shows by where and by how much is the correction applied to account for scattering d) Photographs of the resulting printed constructs after dying them in red. e) Timelapses of a blue dye flowing through the constructs. The scattering corrected tomographic VAM produces an object that matches the geometry and function of the model; while conventional tomographic VAM results in an unintended malfunctioning tube at the center of the construct. f) Fluorescence microscopy image of stained nuclei of cells in the fabricated hydrogels (4.0 million cells mL^−1^).

We use scattering corrected tomographic VAM to fabricate cell‐laden constructs that would be difficult to print otherwise.^[^ ^29^, ^30^, ^31^ ^]^ Bioprinting cm‐scale constructs is challenging because hollow channels must be left open to allow for the inflow of nutrients and oxygen to the cells deep inside the hydrogel.^[^ ^32^, ^33^, ^34^, ^35^ ^]^ For this, we used a complex geometry of a 4‐mm solid core surrounded by four millimetric channels, as shown in **Figure** **4**a. Cell‐laden hydrogels may be highly scattering (Figure 4b).^[^ ^16^ ^]^ For volumetric light‐based biofabrication methods, this constrains cell concentration. The proposed scattering correction spatially redistributes light as it is sent in the tomographic patterns. As shown in Figure 4c, to avoid over‐polymerization and clogging the channels, more light is sent to the fine features of the edges while less light is sent to the bulk of the construct. The overall light dose (19.1 ± 5.2mJcm^−2^, equivalent to 6.4mJcm^−3^) and printing time (36 s) were the same to produce the uncorrected and corrected objects in Figure 4d. Tomographic volumetric bioprinting has been shown to have high cell viability immediately after printing and several days after printing.^[^ ^8^, ^13^, ^16^, ^36^ ^]^ As our proposed scattering correction does not change the required light dose to produce a print, and results in small changes in the local light intensities of the projected patterns, it is not expected that cell viability should be compromised. Correcting for scattering did not seem to affect cell‐viability 1 h after printing, as shown in Figure S9, Supporting Information.

Instead of evaluating the fidelity of the prints (these hydrogels are soft and deform on their weight), we evaluate if all design features are present. The timelapse in Figure 4e and Videos S1 and S2, Supporting Information (Section S9, Supporting Information) show a blue liquid dye as it is pumped through the constructs. Conventional tomographic VAM yielded clogged channels and a void core. This comes from the fact that the correct light distribution does not reach the center of the vial during fabrication. Note that a functional object could not be achieved by using neither a lower light dose (this would produce unclogged channels, but the core would still be void) nor a higher light dose (this would produce a solid core, but channels would still be clogged). Scattering‐corrected Tomographic VAM produced a cm‐scale construct with all four channels unclogged and a solid core in a soft hydrogel loaded with 4 million HEK 293 cells mL^−1^ (Figure 4f). Previous reports have demonstrated the fabrication of similar structures under concentrations of only 10 000 or 1 million cells mL^−1^.^[^ ^8^, ^13^ ^]^ At a concentration of 4 million cells mL^−1^, the scattering mean free path of the resin is *l*_s_ = 3.6 mm. The cell‐laden constructs were printed in vials whose inner diameter is *L* = 13 mm. We must emphasize here that the cell concentration in the gel could potentially be further increased by printing in smaller vials, as what limits tomographic VAM is the ratio *l*_s_/*L*. As detailed in Section S7, Supporting Information, our technique enables printing with similar fidelity than conventional tomographic printers but in materials where the amount of scattering is three times larger.

## 4 Conclusion 

In the present work, we reported on the necessity of characterizing and correcting for light scattering to improve the quality of volumetric 3D printing in complex nontransparent materials. We proposed to make a correction based on a spatial frequency analysis of a stack of images captured with a side‐view camera, perpendicular to the optical axis. Corresponding experimental data reveal the poor transmission of high spatial frequencies due to light scattering by the photocurable resin. Following this image analysis, a numerical correction can be performed to compensate for this frequency‐dependent attenuation by accentuating the features of highest spatial frequencies. The resulting corrected light dose presents an increased contrast compared to the standard binary map conventionally used. Through the printing of several object geometries in two different scattering materials (acrylics and hydrogel‐based resins), we demonstrate that the correction improves printing fidelity and resolution.

The proposed apparatus to characterize light propagation through the resin with the side‐view camera may also be used for other type of correction. As an example, correcting for the exponential decrease of ballistic light is also feasible and can be done in real space with a single image (see Section S5, Supporting Information). Correction for absorption can be applied to a broad class of materials, like non‐scattering but absorptive resins.

In all cases, whatever the correction applied, the method still relies on the projection system and hence the use of a ballistic light as in conventional VAM. This means that printing in opaque materials where light undergoes multiple scattering events may not be feasible even if a strong scattering‐correction is applied, simply because the projected patterns become rapidly random. However, the scattering regime studied here, where the correction shows significant improvements on the printing fidelity, is still very relevant for many interesting materials such as bioresins. Cells suspended in such resins are considered as weak scatterers because of their large size (around 10 micron, i.e., mostly forward scattering) but at high concentration this effect becomes detrimental for tomographic printing. Reaching high concentration is however a necessity if one wants to preserve the viability of the print over time. Here, we show that the scattering correction offers the possibility to increase the cell density in the hydrogel without affecting the printed cellular constructs. Here we show significant improvements using a concentration of 4 × 10^6^ HEK293 cells mL^−1^ when printing centimeter‐scale structures in vials whose inner diameter is 13 mm, which is relevant for maintaining cell viability over time.^[^ ^8^ ^]^ Correcting for scattering did not seem to affect cell‐viability 1 h after printing; however, future studies to assess and reduce the possible cytotoxicity and mutagenicity of light‐based bioprinting methods are necessary. The results presented in this work pushes the applicability of tomographic VAM to highly cell‐loaded hydrogels while keeping its higher printing speed (tens of seconds compared to typically tens of minutes for DLP^[^ ^8^, ^37^ ^]^) and the small amount of photoinitiator needed (0.16 mg mL^−1^ in this work, compared to typical values of 0.5–10mg mL^−1^ for DLP bioprinting.^[^ ^38^ ^]^

Similar corrections could also be applied to other printing technologies, such as xolography,^[^ ^10^ ^]^ two photon fabrication, longitudinal or multi‐axial setups. These corrections could also be used in combination with optical tuning and refractive‐index matching of resins to further improve fidelity.^[^ ^15^, ^16^ ^]^

## 5 Experimental Section

### Volumetric Tomographic 3D Printer

The optical setup for tomographic additive manufacturing is depicted in Section S8, Supporting Information. Blue light from four continuous laser diodes at 405 nm (HL40033G, Ushio, Japan) was condensed into a multimode optical fiber (WF 70×70/115/200/400N, CeramOptec, Germany) by means of aspheric lenses (C671‐TME405, Thorlabs, USA). Light was then collimated by means of two cylindrical telescopes onto a digital micromirror device (VIS‐7001, Vialux, Germany). Light patterns from the DMD were then projected onto the resin by means of lens pair with focal lengths *f*_1_ = 100 mm (AC254‐100‐A‐ML, Thorlabs) and *f*_2_ = 250 mm (ACT508‐250‐A‐ML, Thorlabs). An iris at the common focal plane of the lenses filtered out high diffracting orders from the DMD.

The photosensitive resins were held in cylindrical glass vials (inner diameter 16 mm). These vials were set to turn with a high‐precision rotary stage (X‐RSW60C, Zaber, Canada).

Orthogonally to the optical axis of the printer, red light at 678 nm from a laser diode was used to image the printing process. A lens pair with focal lengths *f*_1_ = 75 mm (AC508‐075‐A‐ML, Thorlabs) and *f*_2_ = 250 mm (ACT508‐250‐A‐ML, Thorlabs) produced an image onto a CMOS camera (ACE ACA2000‐50G, Basler, Germany).

### Acrylate Resins

The photo‐curable resins used in this work were prepared by combining di‐pentaerythritol pentaacrylate (SR399; Sartomer, France) or PRO21905 (Sartomer, France) with 0.6 mm phenylbis (2,4,6‐trimethylbenzoyl) phosphine oxide (97%; Sigma Aldrich, USA) in a planetary mixer (KK‐250SE, Kurabo, Japan). A threshold light dose was necessary to induce solidification of the liquid resin. This threshold depended on the functionality of the resin^[^ ^9^ ^]^ and on oxygen inhibition.^[^ ^39^ ^]^ These resins were highly transparent. To make them scattering, TiO_2_ nanoparticles (<100 nm particle size, 99.5%, Sigma Aldrich, Switzerland) were first diluted in ethanol (99.8%, Fischer Chemical, South Africa) and then added to the resins before planetary mixing. The calculated scattering phase functions for these resins is depicted in Figure S3, Supporting Information. The phase function was calculated using the online tool https://omlc.org. The resins were poured into cylindrical glass vials and sonicated for 15 min to remove bubbles.

### Post‐Processing of Printed Parts

Parts were post‐processed by rinsing them in isopropyl alcohol (99 %, Thommen‐Furler, Switzerland) for 3 min under sonication.

### Hydrogels: Synthesis of Gelatin Methacryloyl

Gelatin (G2500, Sigma‐Aldrich) was used to synthetize gelatin methacryloyl (GelMA) following the protocol in ref. [40]. Then, it was filtered and diluted to a concentration of 8% (w/v) in phosphate buffered saline (PBS, 79382, Sigma‐Aldrich). Lithium phenyl‐2,4,6‐trimethylbenzoylphosphinate (LAP, 900889, Sigma‐Aldrich) was added as photoinitiator to the liquid hydrogel at a concentration of 0.16 mg mL^−1^. The material was then bottled in glass containers (inner diameter = 13 mm) and refrigerated to 4 °C for at least 2 h to let them gelify.

### Cell Culture

Human embrionic kidney 293 (HEK 293) cells were cultured in DMEM–high glucose (D6429, Sigma‐Aldrich) supplemented with 10% fetal bovine serum (FBS, F9665, Sigma‐Aldrich). Cells were incubated in flasks under a humidified atmosphere with 5% CO_2_ at 37 °C.^[^ ^41^ ^]^ To prepare cell‐laden hydrogels, cells were detached with 0.25% trypsin + EDTA (Sigma‐Aldrich) for 3 min followed by DMEM + FBS 10%. Cells were then centrifuged at 2000 rpm for 2 min and resuspended in PBS. The concentration of cells was calculated by counting cells in a Neubauer chamber. The corresponding volume of cell suspension was pipetted into the liquid GelMA hydrogel prior to adding the photoinitiator, and gently agitated for 2 min.

HEK 293 cells are immortalized cells, and as such, were not the most representative cell line to demonstrate functional biofabrication. They were, on the other hand, very representative of light scattering induced from loading a bioink with cells.

### Post‐Processing of Printed Hydrogels

Printed hydrogels were rinsed with pre‐warmed PBS for 15 min at 28 °C. The washing medium was changed every 5 min.

### Imaging of Flow through Hydrogels

Hydrogels were colored by immersing them in Allura Red AC (CAS 25956‐17‐6, Sigma‐Aldrich) in PBS (1 mg mL^−1^) for 5 min. Then, they were rinsed with PBS and immersed in deionized water for photographs. To show the functionality of the hydrogels with hollow unobstructed channels, a dark‐blue suspension was pipetted through the constructs. The suspension consisted of Alcian Blue 8GX (A5268, Sigma‐Aldrich) at 1 mg mL^−1^ in 90% glycerol–10% PBS.

### Fluorescence Microscopy of Hydrogels

Printed hydrogels were rinsed in PBS 1×, stained with DRAQ5 (1:500, ThermoFischer) for 30 min, and rinsed in PBS again. They were then imaged under a confocal fluorescence microscope at 638 nm excitation (SP8, Leica). TrackMate on FIJI was used to count cells in the hydrogel after applying a median filter to the 3D stack.^[^ ^42^ ^]^

### Characterization of the Scattering Profile of Resins

A small amount of the resins (acrylates or hydrogels) was put aside before adding the photoinitiator and poured into 10 mm cuvettes with four polished windows. The cuvettes were placed at the image plane of the printer. Series of patterns were displayed on the DMD while photographs were recorded simultaneously with the orthogonal camera.

### MicroCT Imaging and Assessment of Print Fidelity

Printed objects were imaged under a 160 kV X‐ray transmission tomograph (Hamamatsu, Japan) with voxel sizes of 8.4 μm × 8.4 μm × 8.4 μm. 3D visualizations of the pieces were obtained with Avizo software (ThermoFischer, USA).

Quantitative analysis of 3D scans were performed on ImageJ.^[^ ^43^ ^]^ To quantify print fidelity, the object in the microCT scan data was segmented and binarized using Otsu's thresholding.^[^ ^44^ ^]^ The images of the object were centered around its center of mass and rotated to align them with the orientation of the reference shape. The processed stack of images was then saved and imported into a python code, which automatically computed the intersection over union (IoU) for several affine transformations (excluding shear) of the image. From this, the distribution of IoU indices was obtained for each part. The mean IoU and its standard deviation was reported.

To measure the thickness of the parts, the data from the microCT scan was imported into Python as an array. After thresholding it to remove background noise, the array was binarized. Thickness was measured by counting the number of positive voxels.

### 3D Models

FreeCAD (https://www.freecadweb.org/) was used to design the 3D models for the gears and the vasculature model. 3D models for the Sacré Coeur Basilica were obtained freely from https://www.thingiverse.com.

### Visualization

Plots and figures were produced with Matplotlib,^[^ ^45^ ^]^ ImageJ,^[^ ^43^ ^]^ and the colormaps by Fabio Crameri (Zenodo, http://doi.org/10.5281/zenodo.1243862).

## Conflict of Interest

D.L. and P.D. are shareholders and employees of Readily3D SA. C.M. is a shareholder of Readily3D SA. All the other co‐authors declare no conflict of interest.

## Supporting information

Supporting Information

Supplemental Video 1

Supplemental Video 2

## Data Availability

The data that support the findings of this study are available from the corresponding author upon reasonable request.
