# Supplementary material for: Controlling Light in Scattering Materials for Volumetric Additive Manufacturing
Source: Adv Sci (Weinh). 2022 May 18;9(22):2105144. doi: 10.1002/advs.202105144 (PMC9353445; doi:10.1002/advs.202105144)
Supplement: Supplementary file 1 — Supporting Information [file ADVS-9-2105144-s001.pdf]

Controlling light in scattering materials for volumetric additive manufacturing: Supplementary Information

Jorge Madrid-Wolff[1,†] Antoine Boniface[1,†] Damien Loterie[2] Paul Delrot[2] Christophe Moser[1,*]

[1] Laboratory of Applied Photonics Devices, School of Engineering, Ecole Polytechnique Fédérale de Lausanne, CH-1015, Lausanne, Switzerland
[2] Readily3D SA, EPFL Innovation Park, Building A CH-1015 Lausanne, Switzerland
[†] These authors contributed equally to this work
[*]To whom correspondence should be addressed. e-mail: christophe.moser@epfl.ch

Keywords: *Light-based 3D printing, Volumetric Additive Manufacturing, Bioprinting, Light scattering, Complex materials*

S1: Light patterns computation from the target dose

The computation of the light patterns from the target dose relies essentially on the Radon transform as developed for tomographic imaging. However in the case of printing, the problem to solve is reversed: one has to compute the 2D patterns from the 3D dose whereas in imaging the algorithm aims at reconstructing the 3D object from a set of 2D measurements. Therefore, the starting point is for us the 3D model, i.e. the object one intends to print. (*1, 2*) In practice we do not perform the 3D to 2D transform but rather a sequence of 2D to 1D transforms (cross sections perpendicular to the vial's axis, see Figure S1.b) which is computationally more efficient. These binary sections can be eventually filtered with an experimentally-measured mask to compensate for scattering or attenuation (Figure S1.c) as presented in this manuscript. From this target light dose (here attenuation-correction, see Supplementary S4), the Radon transform is performed. The obtained sinogram is filtered in its Fourier domain to compensate for the oversampling of the low spatial frequencies in the projection space (see Figure S1.d). (*3*) If this filtering operation is not done, the cumulative dose projected into the resin would be blurred and compromise printing resolution (see Figure S1.e). This step generates negative values that cannot be optically implemented. They are set to zero, which creates some artefacts. An optimization using gradient descent is performed to improve the fidelity between the target dose and the sinogram back-projection. A dedicated forward model allows rapid optimization of the projected patterns which offers significant improvement on the resulting dose. The code is written in python and uses the PyTorch library for GPU acceleration.

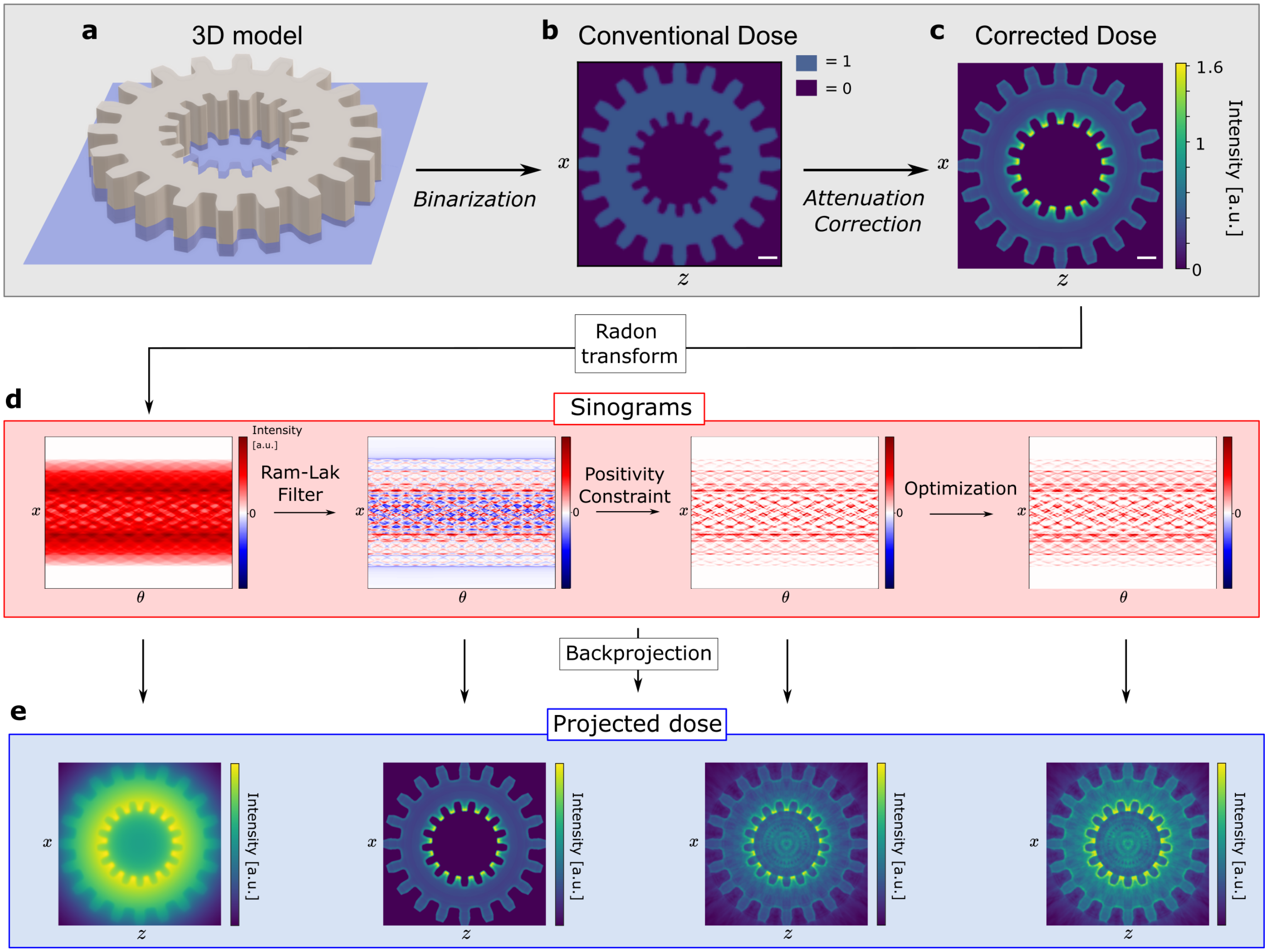

Figure S1: **Procedure to compute the light patterns from the target dose. a.** The 3D model is cut in different slices (**b.**) along the y-axis (vial's axis). A correction can be computed onto this binary dose (here attenuation correction) so that it takes into account the effect of scattering (here, the attenuation of ballistic light). **d.** As in any tomographic volumetric printing technique, we must then compute from the target dose cross section (2D) the set of light patterns (here in 1D). This is achieved by computing the Radon transform. Because the Fourier space is not properly sampled, one needs to apply a Ram-Lak filter. (*3*) The corresponding patterns contain positive and negative values that cannot be optically generated. All the negative values are set to 0 and an optimization algorithm is designed to optimize around this positivity constraint and maximize the dose fidelity. **e.** Corresponding reconstructed dose for the different forward models. Inset: Threshold to mimic the solidification process of the resin.

S2: Computing the scattering correction from experimental data

In Figure S2, we describe in details our method that generates the scattering-corrected light dose. The fact that light patterns get increasingly blurred with depth (see Figure S2.a and different z-profiles in Figure S2.e) is also noticeable in the frequency space (also referred to as “k-space” in the following): the scattering acts as a low-pass filter. In other words, features of high spatial frequencies in the pattern get more rapidly attenuated than the low ones. This is exemplified in Figure S2.b by computing the one-dimensional Fourier transform of Figure S2.a along the y-axis (corresponding profiles at different depths are shown in Figure S2.f). In order to properly characterize the transmission of all the spatial frequencies, a sequence of different patterns (here 100 patterns) is projected onto the DMD. Note that it is important to project patterns whose k-spaces are representative of the frequencies at stake when printing. Suitable sets of patterns include patterns from the Radon transform of the object, random patterns, patterns from the Fourier transform of images or signals, or designed dictionaries of patterns with different spatial frequencies, for example. In Figure S2.c, the average transmission of the spatial frequencies shows the strong attenuation of the high spatial frequencies as light penetrates in the material. To alleviate this unequal impact of scattering in k-space, the amplitude of the frequency components (which are dampened by the scattering) is enhanced. According to these measurements, one can compute a correction mask that ensures to get the amplitudes of all the incident frequency components (at z = 0 mm) constant across the full vial (at all depth z), see Figure S2.d. In practice, this correction mask is obtained by dividing the incident averaged spectrum (k-space domain) at z = 0 mm by each spectrum taken at different z depths. Profiles at different z are plotted in Figure S2.h. They present two symmetric lobes whose amplitude varies with depth. As expected, the correction to apply is more important for high spatial frequencies up to a certain point above which the correction drops, simply because the initial energy (at z = 0 mm) of the corresponding frequencies is very low. These correction masks are 1D whereas the target objects are 2D (slice of the 3D object). Importantly the effect of scattering in k-space should be the same whatever the direction (k_x, k_y or k_z), simply because the TiO_2 particles are homogeneously dispersed in the resin. So there can be easily computed a higher dimensional mask from the curves in Figure S2.h by applying an axial symmetry with respect to the central frequency $k_y = 0$ mm^{-1}, see Figure S2.k. The correction mask is then applied onto the target binary object in Fourier (see Figure S2.j). Different filtering masks are computed depending on the depth at which the correction is performed. The longer the distance over which light must travel in the scattering resin the stronger is the correction to be applied (see Figure S2.l).

In our tomographic system, the penetration depth increases radially and is maximal at the center of the vial (i.e. 8 mm in our case), because of the rotation. This region is, in such apparatus, the one where the scattering of light causes the most difficulties and therefore where the correction must be the most important. Because the correction in k-space is depth-dependent, they are valid for restricted regions that look like annulus (see Figure S2.m). Their radius are connected to the depth at which the correction is done and their thickness gives the accuracy of the reconstruction. For sake of clarity, the depth discretization is of 500 micrometers but a step of 100 micrometers is preferably taken in practice. In Figure S2.n we represent the resulting scattering corrected light dose, which is the sum over all the annular dose distributions. Compared to the binary map, conventionally use for computing the patterns, this corrected target obtained from experimental measurements has a much higher contrast, especially when moving towards the center. The latter is then used as target light dose (instead of the binary map Figure S2.i), to compute the scattering corrected light patterns.

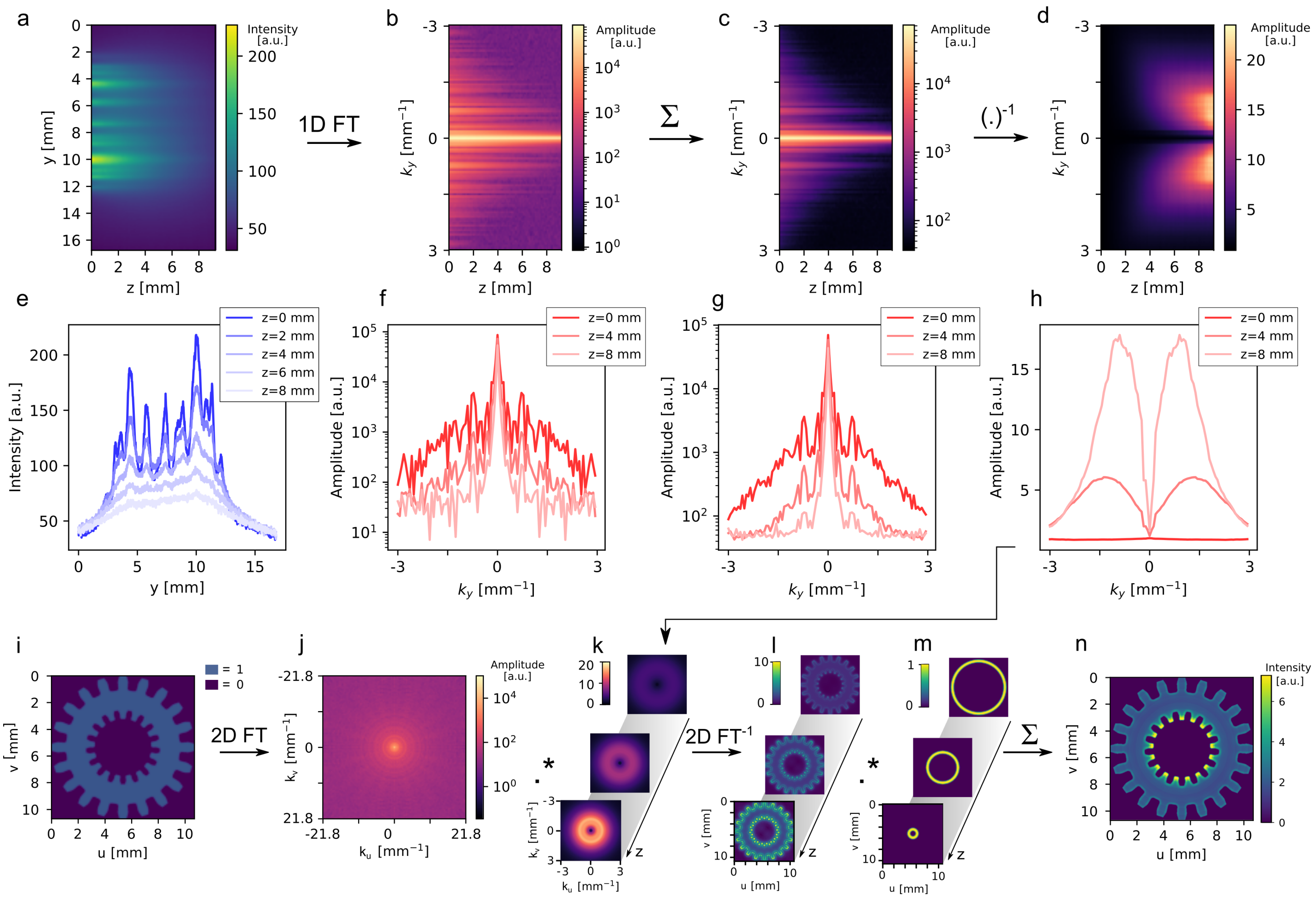

Figure S2: **Scattering correction workflow**. **a.** Typical side-view image. **b.** Corresponding one-dimensional Fourier transform of the pattern along y-axis as a function of penetration depth. **c.** Sum over a set of different projected patterns (here 100 patterns) **d.** Correction needed for maintaining a uniform energy for all the spatial frequencies at all depths. **e.-h.** Cross sections of the corresponding above images for different depths. **i.** Binary target 2D object to print. **j.** Amplitude of the corresponding 2D Fourier transform **k.** Two-dimensional masks obtained from the interpolation (in polar coordinates) of the curves in (h.) **l.** Inverse two-dimensional Fourier transform of the filtered space. **m.** Each filtering is valid only for a given depth, corresponding to a specific annulus in polar coordinates. **n.** Resulting scattering corrected light dose.

S3: Optical properties of the scattering acrylates

In this section we provide additional information on the scattering properties of our scattering acrylates. The scattering resins are prepared in the following manner: a transparent resin (here, Dipentaerythritol Pentaacrylate, SR399, Sartomer Arkema) is made scattering by adding TiO_2 nanoparticles (TiO_2 nanopowder, < 100 nm particle size, Sigma Aldrich) homogeneously dispersed in the resin. The concentration of TiO_2 is around 0.3 mg/mL. Although this concentration is low, the scattering induced by TiO_2 nanoparticles is very high (refractive index of TiO_2 is 2.9 at 400 nm compared to 1.5 for the monomer). Knowing the size of the particles and the refractive index mismatch we can derive from Mie scattering formulas the theoretical phase function (open source calculator: https://omlc.org/). (*4*) This reveals the relatively high isotropy of scattered light, see Figure S3.a. It translates into a low anisotropy factor $g = 0.39$ (average cosine of phase function). Experimentally, from the ballistic light exponential decay we can retrieve the scattering mean free path of light, l_s. Figures S3.b-c (respectively Figures S3.d-e) correspond to the scattering resin used to print the gear in Figure 3.a (the Sacré Coeur Basilica in Figure 3.b). From the scattering mean free path, one can estimate the transport mean free path $l_t = l_s/(1 - g)$

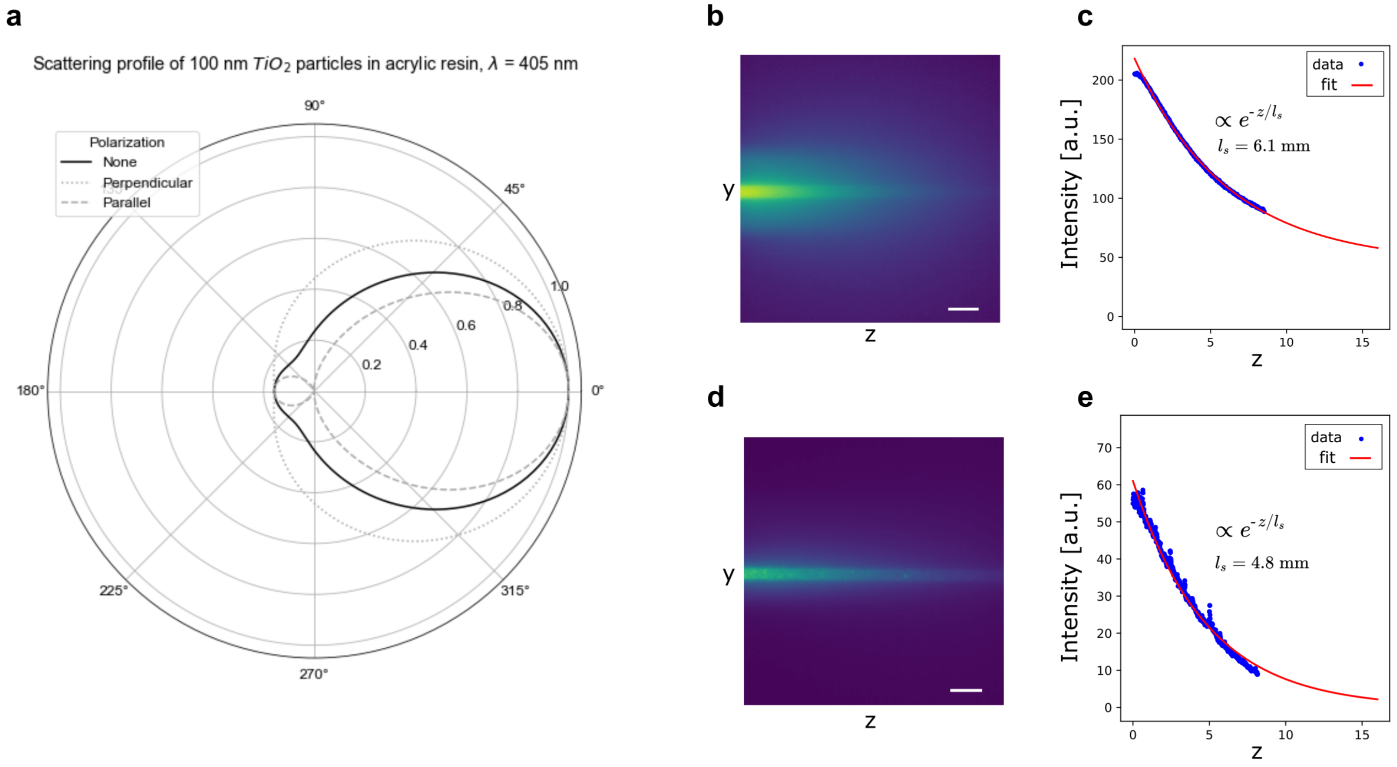

Figure S3: **Optical properties of the scattering resins. a.** Phase function of TiO_2 at $\lambda = 405$ nm. **b.** Side-view image of a thin sheet of light through the resin used in Figure 3.a. **c.** Exponential decrease of ballistic light. **d.** Side-view image of a thin sheet of light through the resin used in Figure 3.b. **e.** Exponential decrease of ballistic light.

S4: Scattering correction effect on the projected patterns

We use the 3D model of the non-axially symmetrical Sacre Coeur Basilica to exemplify our proposed correction results in the spatial rearrangement of light. The correction applied onto the target 3D dose is done following the same protocol as in Figure S2. A cross-section along the x-axis (indicated in Figure S4.a) provided in Figure S4.b shows the importance of depositing more light on the edge of the object (regions of high spatial frequency) which increases the contrast of the target dose and thus compensate for the scattering effects that tends to smooth the print. One can also notice the correction ensures to bring more light close to the middle of the vial (indicated by vertical dashed white lines) to take into account the attenuation. This correction onto the target dose translates in the set of projected patterns to print. In Figure S4.c we represent the light patterns at $\theta = 0°, 45°$ and $90°$ and shows the induced correction by computing the difference map (see Figure S4.d). Figure S4.e and f, are photographs and x-ray scans of the obtained print, respectively. As observed with the gear in figure 3, light scattering prevents from printing the full object without over-polymerizing parts of the structures, mainly the ones closer to the edge of the vial. For instance here, the main square tower cannot be printed correctly. This issue does not occur when the scattering is taken into account prior to computing the light patterns. Also, the correction offers a significantly better resolution to the resulting print. Naturally, even with scattering correction, printing in scattering resin has some limitation and the resolution of analogous structures in transparent resins cannot be achieved. Here, the correction aims at depositing more or less light, knowing in advance how light intensity is scattered on average. However, a finer characterization of the scattering process using a laser, including the distortion of its phase for instance, might provide a better correction and would potentially allow, using wavefront shaping techniques, for improving the printing fidelity.

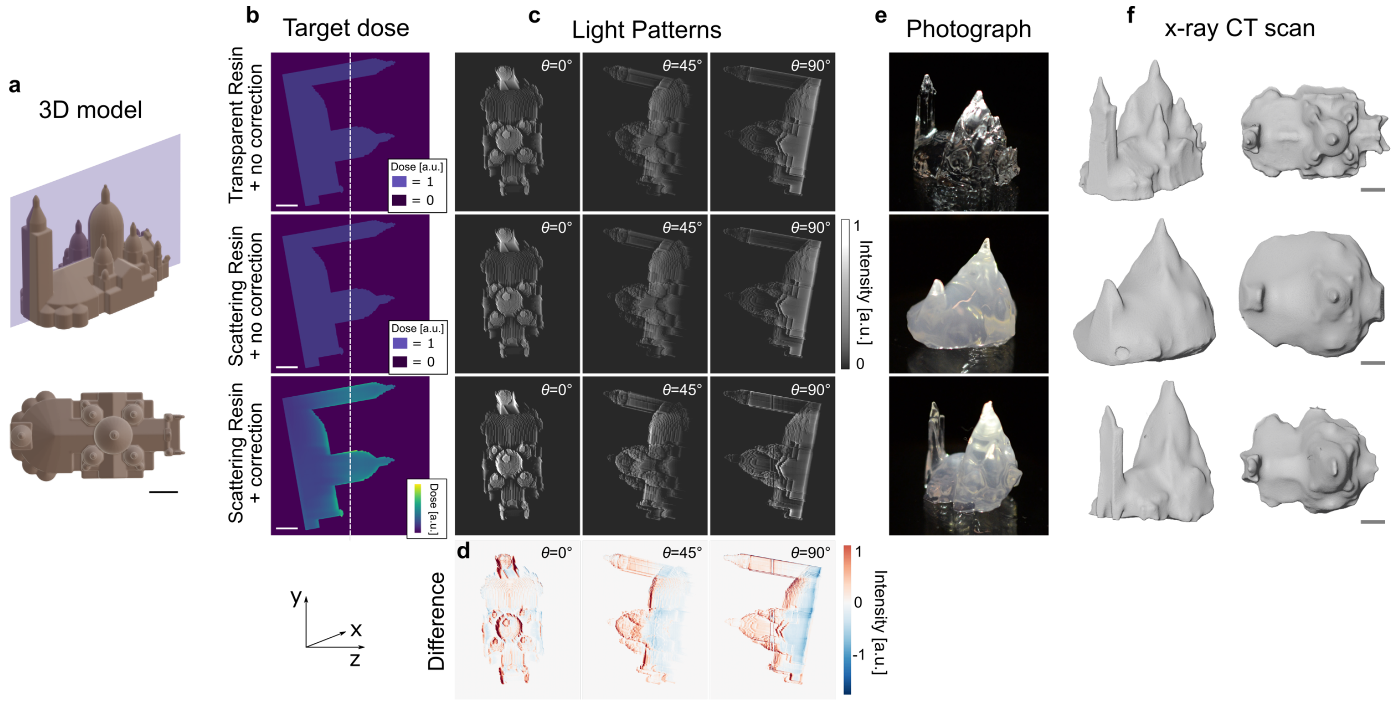

Figure S4: **Volumetric digital manufacturing in scattering resins**. **a.** 3D model of the Sacré Coeur basilica in Paris. **b.** Target dose to polymerize the resin. Top and Middle panel: binary mapping (no correction), Bottom panel: Scattering-corrected target dose. **c.** Corresponding exemplary projected patterns to polymerize the resin for different angles. **d.** Difference between bottom and middle line to visualize the resulting correction. **e.** Photographs of the printed shapes. In this experiment the scattering mean free path is ($l_s = 4.8$ mm, $l_t \simeq 7.9$ mm) **f.** Oblique and top views from a x-ray micro Computer Tomography (CT) scan of the resulting objects. (Scalebars: 2 mm).

S5: Attenuation correction

A major consequence of light scattering is the exponential decrease of ballistic light intensity. This phenomenon can be easily registered and analysed using a detection orthogonal to the optical axis (with the side-view camera presented in Figure 2 and Supp. Figure S12). In Figure S5 we propose a procedure (similar to Figure S2) to correct for this exponential attenuation of ballistic light. The measurement consists here of sending through the resin a relatively narrow laser beam (in both x and y directions). In practice, this is achieved by activating only a few pixels on the DMD.

The amount of ballistic light as a function of depth is characterized by measuring the intensity decrease along the z-axis. Experimental data are reported in Figure S5.b and fitted with a negative exponential. Fitting coefficients provide an estimation of the scattering mean free path l_s, which is the average mean free path length between two successive scattering events. The fit reported in Figure S5.b gives $ls = 7.3$ mm. The cuvette used for the measurement is only 10 mm thick but the fit allows extrapolating the trend across the entire vial (cylindrical 16 mm diameter reservoir used for printing). In tomographic volumetric printing, the vial (i.e. the resin container) rotates with respect to its center, and it is thus necessary to consider the total amount of light deposited inside after a full rotation (360°). In particular after half a turn a similar exponential decrease of ballistic light would be observed, because the TiO_2 particles are dispersed uniformly inside the resin. So in average the amount of ballistic light in the vial is the sum of the two negative exponential curves (bold-line curve and its symmetric with respect to the axis of rotation at z = 8 mm in dashed). Because the decrease is exponential, the amount of light in the vial is not uniform after a full rotation. In particular, the proportion of ballistic photons is smallest at the center of rotation of the resin container. Here for the scattering resin under study, there is, in average, 40% less light in the

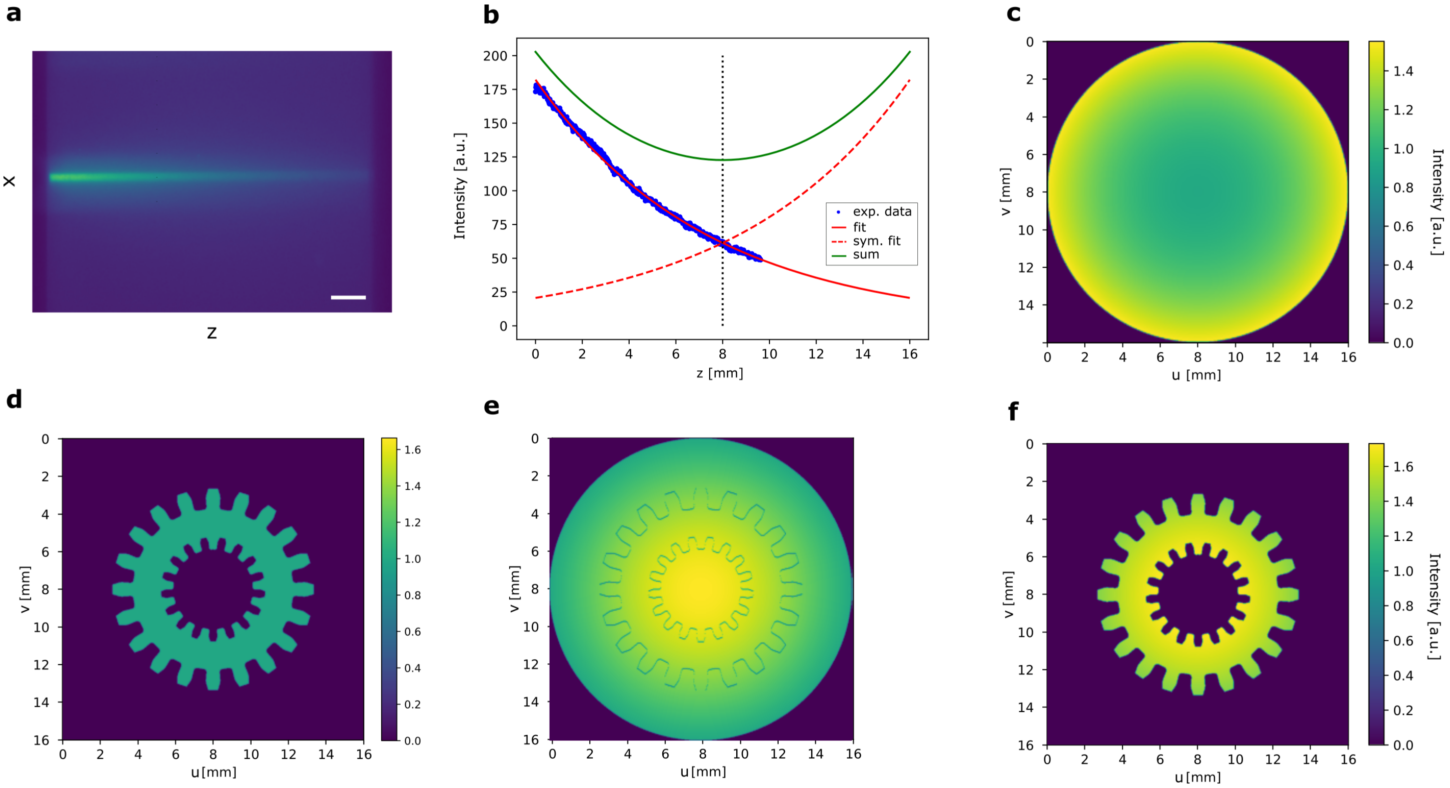

Figure S5: **Attenuation correction of light. a.** Typical side-view image captured on the camera perpendicular to the optical axis. A thin sheet of light is sent through the scattering resin for characterization. Scale bar = 1 mm. **b.** Experimental data are fitted with a negative exponential. After half a turn the equivalent decrease can be observed from the other side (represented by the symmetrical with respect to the center of the vial at z = 8 mm, dashed red curve). The cumulative dose inside resin after 360° is the green curve (i.e. the sum of the two red curves). In average there is a lack of light in the middle of the vial. **c.** Two-dimensional map of the light amount in the vial (top view). **d.** Binary mapping of the object to print. **e.** Two-dimensional correction mask. **f.** Attenuation-corrected light dose.

middle of the vial (i.e. after 8 mm of propagation) than on the edges. In Figure S5.c the corresponding two-dimensional profile of ballistic light across the vial (16 mm in diameter) is reconstructed. The latter is obtained by interpolating the bold-line curve reported in the plot of Figure S5.b in polar coordinates. This two-dimensional map is then inverted and gives access to what is designated herein as "correction mask" (Figure S5.e). To account for the exponential decrease of ballistic light inside the scattering resin, this correction mask is applied onto the binary map (Figure S5.d) prior to computing the light patterns with the Radon transform. The resulting light dose to project inside the resin to print, taking into consideration the attenuation of ballistic light, is represented in Figure S5.f. It corresponds to the product of the binary map with the correction mask.

Note that here the correction compensates for the exponential decrease of ballistic light due to scattering resin but the effect of pure sample absorption can be treated similarly. A useful application would be to correct light absorption from dyes or one or more photoinitiators. Absorption from photoinitiators is essential to polymerize the resin, but it limits the performance of the printer, such as for resolution or print size. Usually, the concentration of one or more photoinitiators is chosen so that absorption is very small across the vial, but this means that more light (i.e, more time) is needed to print. Also, correcting for the absorption one or more photoinitiators offers the possibility to print faster, to print in weakly polymerizing or crosslinking materials, or to produce larger objects.

S6: Correction impact on the printing process

To demonstrate that the applied correction changes notably the printing process, we image with the side-view camera images the vial under red light exposure at different times of the polymerization, as shown in Figure S6.a. These videos prove that the correction takes well into account the effect of scattering. If no correction is applied (patterns computed from the binary target dose in S6.b), the object builds from the edge of the vial, where it receives more light. This means printing in the center of the vial requires longer exposure which inevitably over-polymerizes the parts of the object close to the vial's edge. Here, the positioning of the print induces over-polymerization of the basilica's floor as one can see in Figure 3. The correction prevents from printing the structure radially from the edge to the center of the vial. In Figure S6.e, images acquired at the beginning of the polymerization shows that the dome of the basilica as well as its floor appear at the same time. It demonstrates that the correction apply to deposit more light in the central region of the vial works which strongly limits the over-polymerization of parts of the object.

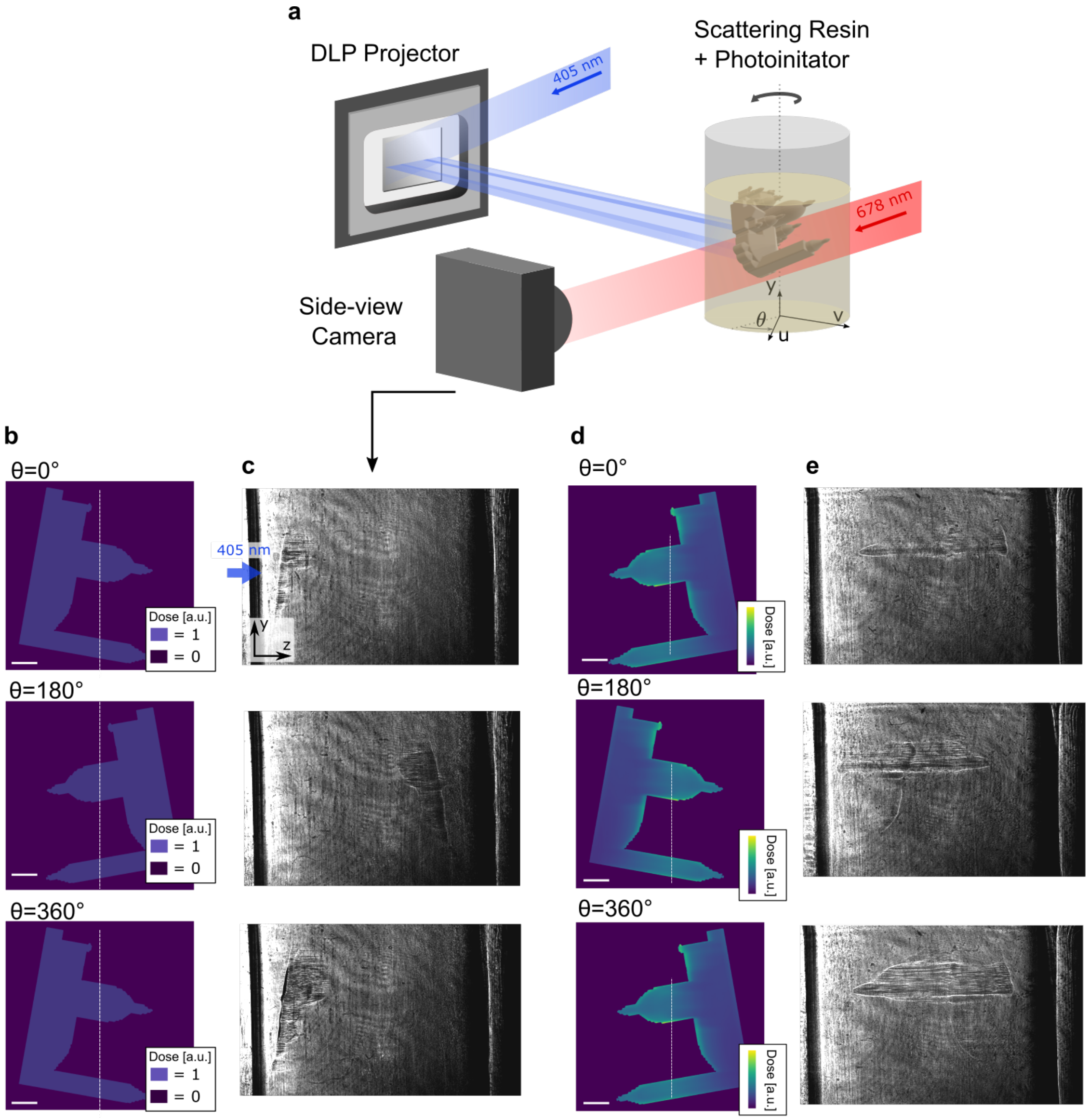

Figure S6: **Real-time monitoring of the printing with and without corrected patterns. a.** Schematic view of the printer with the passive monitoring arm. **b.** Non-corrected target light dose used to compute the pattern. **c.** Images acquired on the side-view camera during printing in a scattering resin. When no correction is applied the polymerization starts close to the vial's edge. **d.** Scattering-corrected target light dose used to compute the pattern. **e.** Images acquired on the side-view camera during printing in a scattering resin. The applied correction also modifies the printing process.

S7: Printability enhancement using scattering-corrections

In the following, we provide a qualitative study on the printability in different scattering regimes with and without the proposed scattering correction. The amount of light scattering is generally characterized by the scattering mean free path, denoted l_s. It physically represents the average mean free path length between two successive scattering events and is reached when the amount of ballistic component of light, denoted $I_{z=l_s}$, is a fraction of $e^{-1} \approx 37\%$ of the incoming light. In the case of tomographic VAM printing, this parameter is not sufficient to well characterize the system's printability in general. The vial's diameter L (along with the light patterns propagate) is also a parameter to consider as for a given l_s the larger L is, the more difficult it is to transmit light, thus printing with high fidelity. Hence, we report on the ratio L/l_s that best takes into account the effect of light scattering in tomographic printing.

It was previously reported that a conventional tomographic printer works well only if the vial's diameter L does not exceed the penetration depth. (*5*) Originally, this was first investigated to determine the maximal concentration of photoinitiator (resulting in a maximal absorption of light within the build resin) using the Beer-Lambert law. In this case, it was found that the absorption length, l_a often referred to as the penetration depth, should be at least $l_a > L$. If we translate this absorption analysis to scattering that also follows an exponential decay (more precisely the ballistic component of the incoming light is exponentially attenuated with depth), we can state that a first requirement for a scattering resin to be tomographically printed is $L/l_s > 1$. It is important to emphasize here that this is an idealistic case where absorption from the photoinitiator is neglected as well as the spatial distortion of the light patterns that naturally occurs in scattering materials, preventing from high resolution printing. So in practice, we expect this upper bound to be even lower.

If $L/l_s > 1$ a conventional tomographic printer does not perform well. Additional scattering corrections like the one presented here are necessary to extend the printability of tomographic printing in this regime. As reported in our work, the implementation of the scattering correction allows printing in cell-laden hydrogels in vials whose inner diameter is $L = 13$ mm with a scattering length of $l_s = 3.6$ mm which represents a ratio of $L/l_s \approx 3.6$. We also printed in scattering acrylates with high fidelity in vials whose inner diameter is $L = 15$ mm with a scattering length down to $l_s = 4.8$ mm which represents a ratio of $L/l_s \approx 3.1$. With these two experiments in two different scattering materials, we can claim that our scattering correction enables to extend the capabilities of conventional tomographic printing from $L/l_s \approx 1$ to $L/l_s > 3$. This is further illustrated in the figure below.

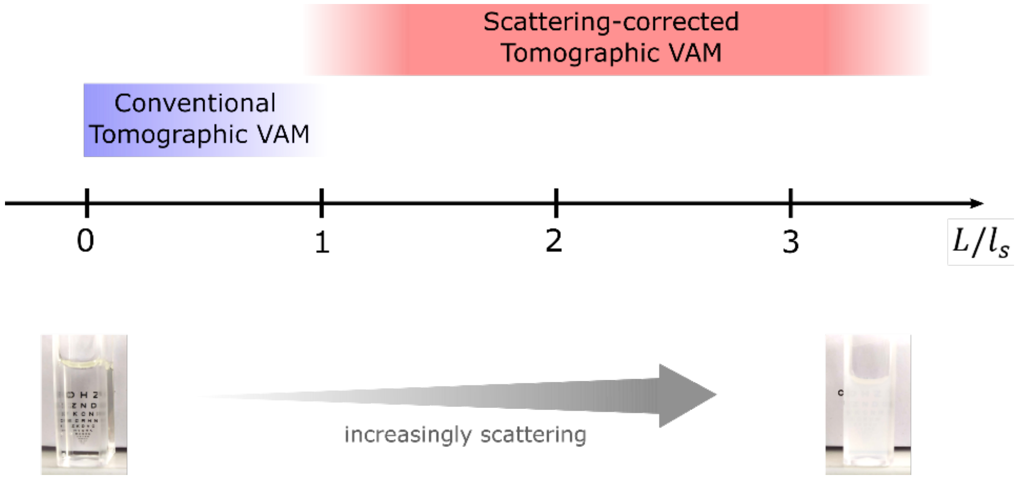

Figure S7: Performance of conventional and scattering-corrected tomographic VAM in different scattering regimes.

S8: Cell-laden hydrogels

Choice of hydrogel.

In this work we used GelMA 8% (w/v) with Lithium phenyl-2,4,6-trimethylbenzoylphosphinate (0.16

mg/mL) as photoinitiator. As reported by Gehlen *et al.*, it is expected that the concentration of initiator affects print fidelity. (*6*) Additionaly, alternative photoinitiators that absorb longer wavelengths, such as the Ru/SPS system introduced by Lim *et al.*, (*7, 8*) could also be used in Tomographic VAM to reduce scattering. They have already been used by Kelly *et al.* to polymerize GelMA hydrogels and by Bernal *et al.* to post-cure tomographically printed cell-laden hydrogels. (*1, 5*). Another interesting approach is that of Bernal *et al.*, in which the contrast agent iodixanol is included in the hydrogel to reduce the refractive index mismatch between the cells and the surrounding hydrogel. (*5*) This strategy had previously been demonstrated by Boothe *et al.* for applications in microscopy and imaging, without compromising viability.

Note that all these strategies (using longer wavelengths for photopolymerization or reducing refractive index mismatch) aim at increasing l_s. Still, scattering will be present to some degree in these materials. The method we propose here is not rival but complementary to these strategies, and could allow to use light-based volumetric additive manufacturing to new families of resins.

Light intensities and phototoxicity

Phototoxicity is a relevant concern to light-based bioprinting technologies. Other works have reported high cell viability after tomographic printing. Bernal *et al.* showed viability $> 85\%$ after printing for equine-derived articular cartilage progenitor cells in 10% GelMA with 0.037% (w/v) LAP. (*5*) Rizzo *et al.* showed high cell viability ($> 90\%$) after bioprinting with mouse myoblasts (C2C12) and normal human dermal fibroblasts (NHDF) over 1 week of culture in both 5% and 2.5% Gel-NB/PEG4SH resin. (*9*) Gehlen *et al.* explored the effect of LAP concentration on the viability of Human Mesenchymal Stem Cells (hMSC) printed in 5% GelMA. They showed that 7 days after printing, cell viability was high ($> 92\%$) for all concentrations of LAP (0.03%, 0.05%, and 0.08%). They do report changes in cell morphology for the higher LAP concentration. (*6*) In this work, we used an LAP concentration of 0.016% (w/v).

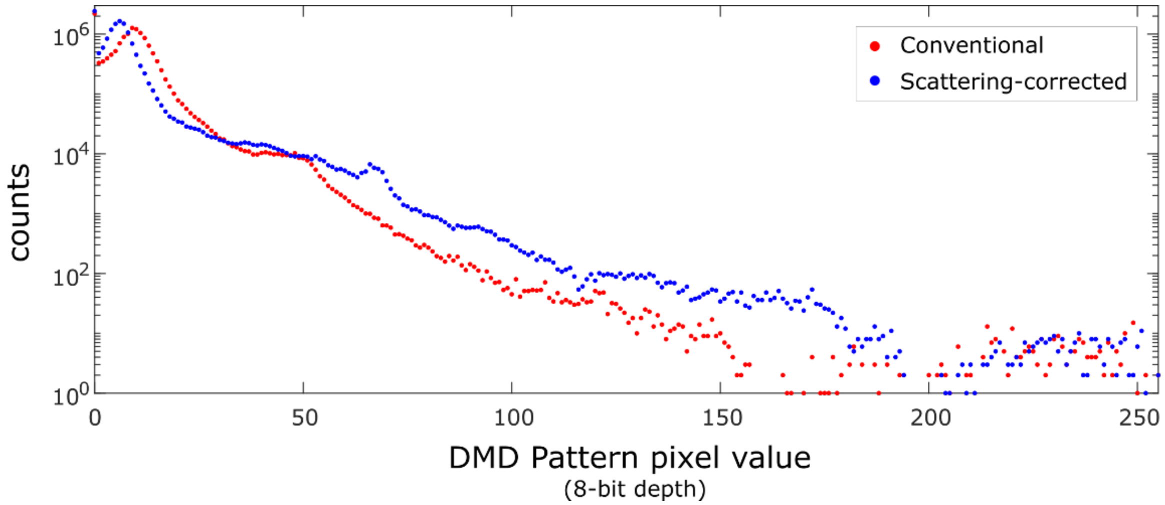

Figure S8: Distribution of light patterns pixel intensity values (8-bit grayscale unit) projected onto the DMD and used for printing the 3D vasculature-channel model with conventional and scattering-corrected tomographic approaches. Y-axis in logarithmic scale.

A question that arises is whether this still holds when a scattering correction is applied. To answer, we need to list and quantify the differences between conventional and scattering-corrected tomographic VAM. In the experiment reported in Figure 4 of the manuscript, the structures were printed with Conventional and Scattering-corrected tomographic VAM in the same hydrogel formulation (same concentration of cells, gelatin, and photoinitiatior). Additionally, they were illuminated in average with very similar light intensities ($0.40mW/0.75cm^2 = 0.53mWcm^{-2}$) and during the same time (36 seconds) which represents a light dose of $19.1 \pm 5.2mJcm^{-2}$ (equivalent to $6.4mJcm^{-3}$, assuming light follows the Beer-Lambert law). In both cases, the average amount of light sent onto the resin is comparable and the only difference lies in the spatial distribution of the light patterns. For instance, if we take the 3D model of the vasculature, we note that the scattering corrected computed light dose (grayscale values) exhibits more light on the edges of the structure, especially on the walls of the inner channels, as seen in Figure 4c. The gel will scatter

these projected corrected light patterns in such a way that the pre-compensation will produce a uniform light dose deposition inside the gel.

Scattering correction results in patterns with higher contrast, as seen in figure S8. The fact that the patterns are locally more intense (up to 1.6 times) may potentially impact the cells, but only the ones close to the edge of the vial, where light is not yet scattered. Fortunately, in a tomographic printer, the vial containing the cell-laden resin rotates (at 30 deg/s) which means that these cells are only illuminated for a very short time. If we consider our experiment (vial's inner diameter = 13 mm), a cell (typical size = 10 um) on its edge would be exposed to this high intensity during 3 ms. At these light intensities, We do not expect that a local difference ($< 60\%$) in intensity would produce any significant effect over 3 ms on cell viability of the construct. Therefore, we can expect that cell viability post fabrication with VAM scattering correction pattern could be comparable to cell viability obtained with conventional VAM.

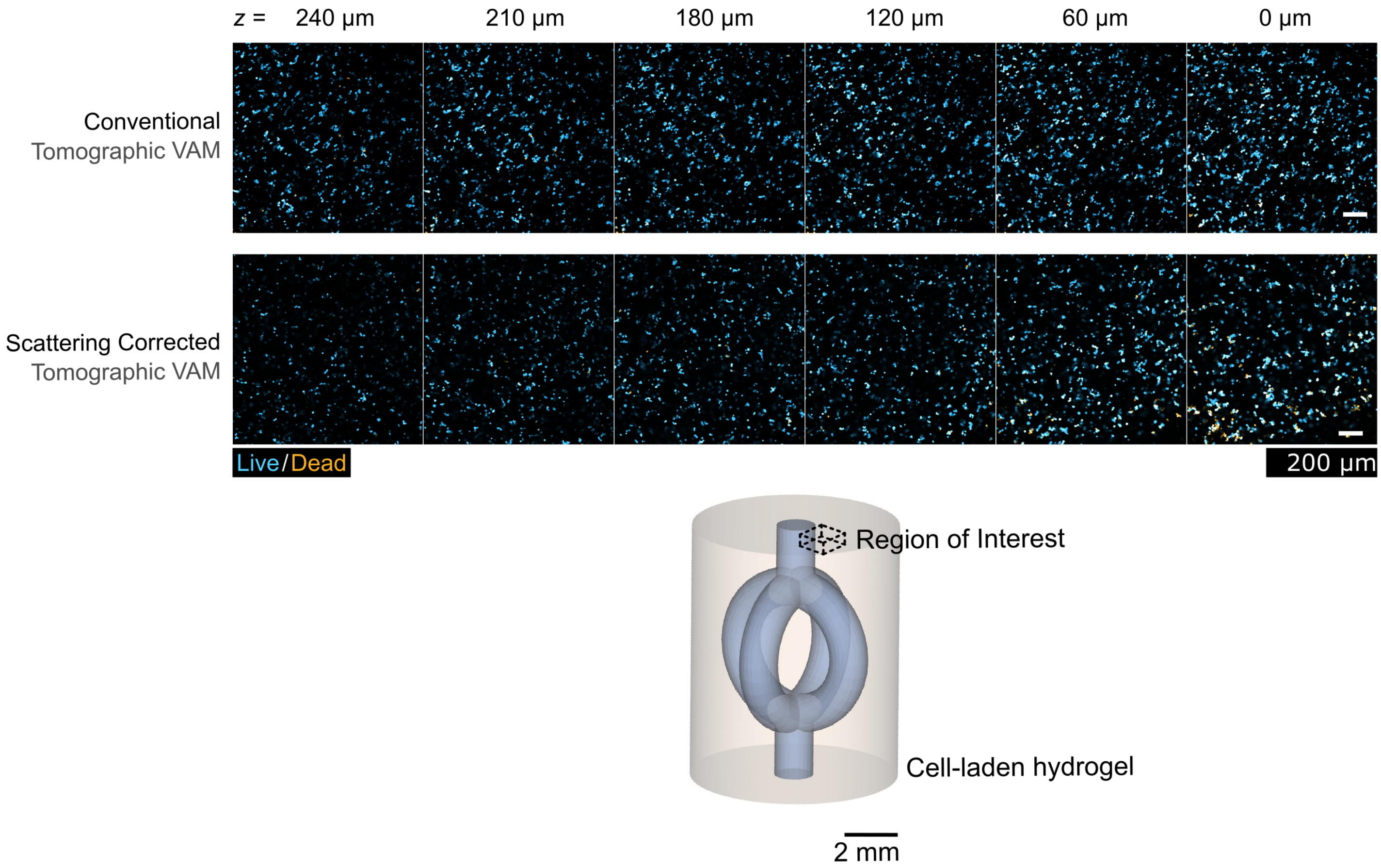

Figure S9: Cell Viability 1h after printing for hydrogels printed with conventional and scattering-corrected Tomographic VAM under the same conditions as in Fig. 4. Cells were labeled with Calcein AM (live) and Propidium Iodide (dead). Hydrogels were imaged at different depths from the surface.

We evaluated cell viability 1h after printing by means of a Live/Dead fluorescence assay. GelMA hydrogels containing 4 million HEK 293 cells mL^{-1} were printed with and without scattering corrections. The constructs were gently washed in pre-warmed PBS at 28°C. They were then post-cured for 2 minutes under a blue LED ($\lambda = 410$ nm) in in RU/SPS (0.2 μM/2 μM) in PBS. The stiffer hydrogels were rinsed twice in PBS and reimmersed in DMEM without phenol red. Then, the constructs were stained with Calcein, AM (3 μM) and Propidium Iodide (5 μM) in PBS at room temperature under gentle agitation for 1 hour in the dark. They were rinsed three times with PBS before imaging them under an inverted Leica SP8 confocal microscope. The constructs were imaged immersed in PBS in a 24-well Corning plate. Calcein, AM was excited using a laser at $\lambda = 488$ nm, and its emission was collected between $\lambda = 500$ and 545 nm. Propidium iodide was excited using a laser at $\lambda = 552$ nm, and its emission was collected between $\lambda = 600$ and 650 nm. Imaging was done using a 10x/NA=0.30 HC PL Fluotar air objective. Stacks of

images at different depths were collected with a slicing of 20 μm. The dual-channel images were produced using ImageJ and the LUTs of Christophe Leterrier (https://github.com/cleterrier/ChrisLUTs).

A qualitative analysis of the images from the Live/Dead assay shown in Sup. Figure S9 show that there is no striking difference between the hydrogels printed with conventional and with scattering-corrected tomographic VAM. Most cells were alive after the printing, and there are no spatial differences in the amount of live cells. These preliminary results go in line with our analysis of light intensities shown in Fig. S8. More detailed studies on the effect of phototoxicty on bioprinted constructs are necessary to better understand the biological constrains of light-based bioprinting.

S9: Supplementary videos

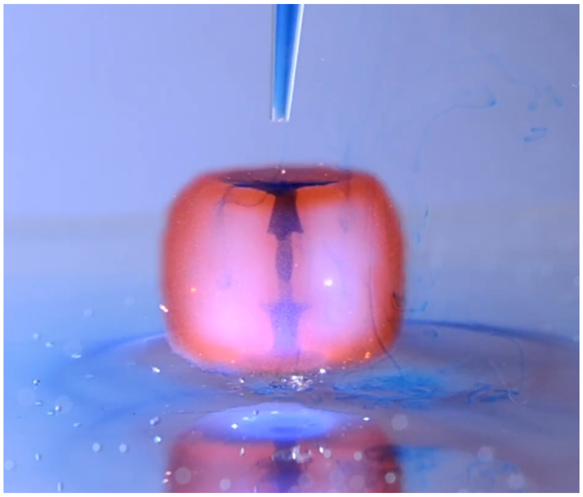

Figure S10: *Snapshot of supplementary video 1.* **Visualization of printed channels in cell-laden hydrogel with conventional tomographic VAM.** The video shows a liquid blue dye being perfused through the resulting single channel of the print.

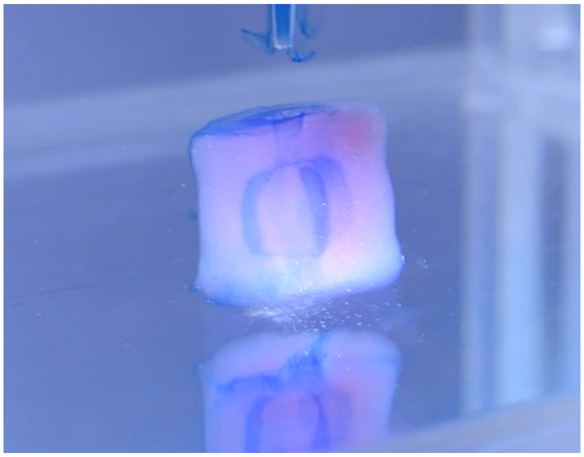

Figure S11: *Snapshot of supplementary video 2.* **Visualization of printed channels in cell-laden hydrogel with scattering-corrected tomographic VAM.** The video shows a liquid blue dye being perfused through the resulting 4 channels surrounding the core of the print; which match the target geometry from Figure 4.

S10: Optical setup

The optical setup for the tomographic volumetric 3D printer is presented in figure S12. The same setup is used to characterize the scattering properties of the resins.

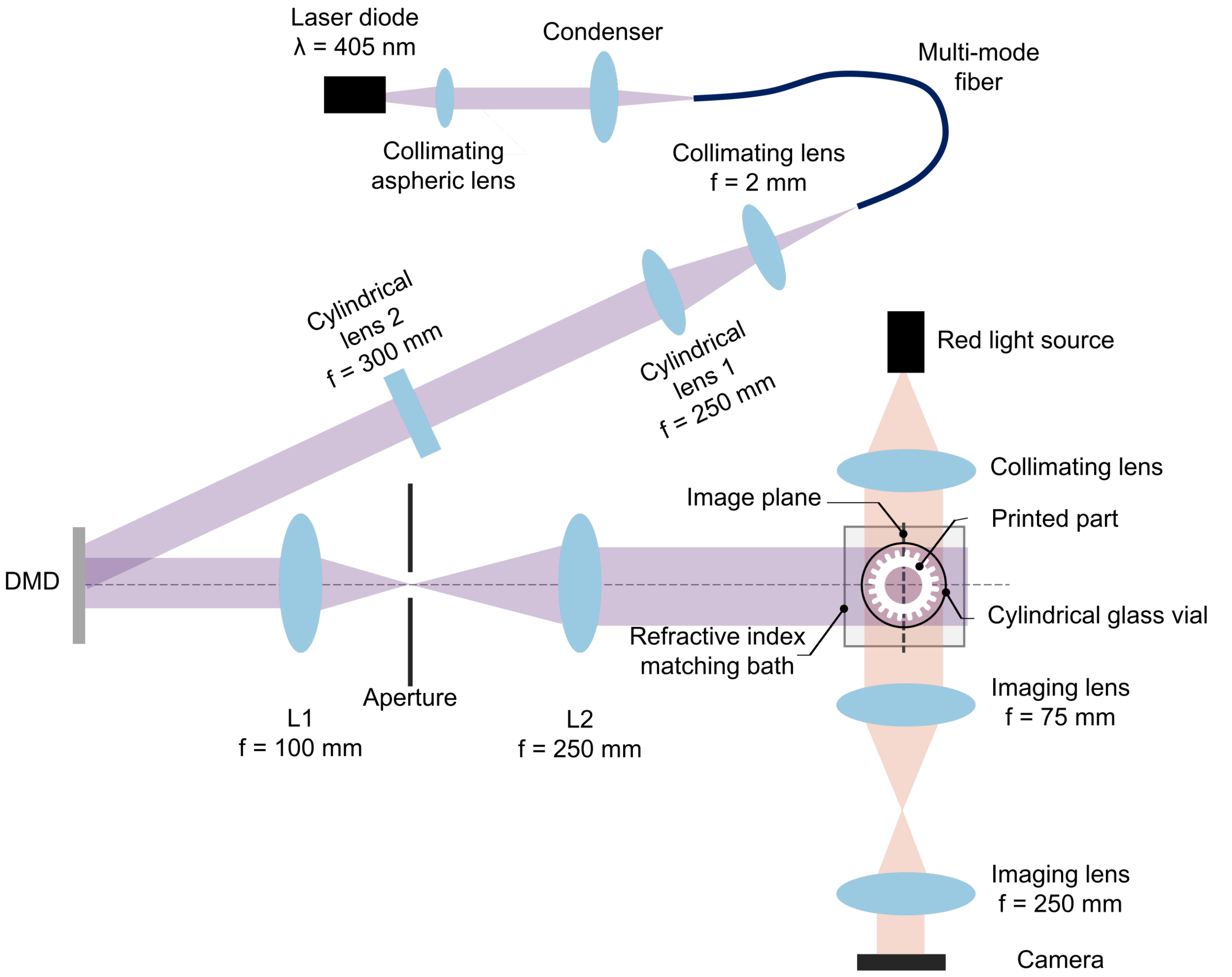

Figure S12: Optical setup of the tomographic volumetric 3D printer.

References

1. B. E. Kelly, I. Bhattacharya, H. Heidari, M. Shusteff, C. M. Spadaccini, and H. K. Taylor, “Volumetric additive manufacturing via tomographic reconstruction,” Science, vol. 363, no. 6431, pp. 1075–1079, 2019.

2. D. Loterie, P. Delrot, and C. Moser, “High-resolution tomographic volumetric additive manufacturing,” Nature Communications, vol. 11, no. 1, pp. 1–6, 2020.

3. A. C. Kak, M. Slaney, and G. Wang, “Principles of computerized tomographic imaging,” 2002.

4. S. L. Jacques, “Optical properties of biological tissues: a review,” Physics in Medicine & Biology, vol. 58, no. 11, p. R37, 2013.

5. P. N. Bernal, P. Delrot, D. Loterie, Y. Li, J. Malda, C. Moser, and R. Levato, “Volumetric bioprinting of complex living-tissue constructs within seconds,” Advanced Materials, vol. 31, no. 42, p. 1904209, 2019.

6. J. Gehlen, W. Qiu, R. Müller, and X.-H. Qin, "Volumetric tomographic 3d bioprinting of heterocellular bone-like tissues in seconds," bioRxiv, 2021.

7. K. S. Lim, B. S. Schon, N. V. Mekhileri, G. C. Brown, C. M. Chia, S. Prabakar, G. J. Hooper, and T. B. Woodfield, "New visible-light photoinitiating system for improved print fidelity in gelatin-based bioinks," ACS Biomaterials Science & Engineering, vol. 2, no. 10, pp. 1752–1762, 2016.

8. K. S. Lim, B. J. Klotz, G. C. Lindberg, F. P. Melchels, G. J. Hooper, J. Malda, D. Gawlitta, and T. B. Woodfield, "Visible light cross-linking of gelatin hydrogels offers an enhanced cell microenvironment with improved light penetration depth," Macromolecular Bioscience, vol. 19, no. 6, p. 1900098, 2019.

9. R. Rizzo, D. Ruetsche, H. Liu, and M. Zenobi-Wong, "Optimized photoclick (bio) resins for fast volumetric bioprinting," Advanced Materials, p. 2102900, 2021.
